# Differential response pathways of *Picea asperata* seedlings from different provenances to altitudinal transfer

**DOI:** 10.3389/fpls.2025.1679777

**Published:** 2025-11-07

**Authors:** Jiangkai Xie, Jiayi Deng, Tairui Liu, Jinping Guo, Yunxiang Zhang, Meng Yang

**Affiliations:** 1 Guangxi Key Laboratory of Forest Ecology and Conservation, College of Forestry, Guangxi University, Nanning, China; 2 Guangxi Colleges and Universities Key Laboratory for Forestry Science and Engineering, College of Forestry, Guangxi University, Nanning, China; 3 College of Forestry, Shanxi Agricultural University, Jinzhong, China

**Keywords:** elevation, transplantation, *Picea asperata* seedling, seedling physiological function, nutrient traits, morphological traits

## Abstract

In mountain ecosystems, the native altitude acclimation and transplantation altitude response strategies of plant seedlings may provide theoretical guidance and strong evidence for addressing the continuous reduction of species' suitable habitats caused by global changes. However, our understanding of the adaptation to native altitude, altitude gradient responses, and underlying mechanisms of native mountain tree species in North China is still unclear. We designed a field experiment in mountainous areas where seedlings from different provenance altitudes (low altitude: 1600 m; high altitude: 2400 m) were transplanted to four typical altitudes. By measuring 18 functional trait indicators related to physiology, leaf characteristics, and nutrients, we attempted to reveal the adaptation of *Picea asperata* to native altitude and the differential responses and mechanisms to altitude changes. The results showed that: (1) Native altitude regulated the seedling's photosynthetic strategy (Pn), water strategy (WUE, gsw), morphological strategy (SLA), and nutrient storage (N), but did not affect leaf structure (AvgPA, AvgSL, AvgSW) or carbon storage; (2) Seedlings adapted to altitude changes by altering nutrient storage (NSC, Sugar, Protein) and leaf morphology (AvgPA, AvgSL, AvgSW, SLA); (3) Low-altitude seedlings of *Picea asperata* exhibited environmental dynamic plasticity and achieved coordinated growth of physiological functions, leaf morphology, and carbon storage at 1900 m (the optimal altitude); (4) High-altitude seedlings showed advantages in their native environment, but their adaptability decreased with decreasing transplantation altitude, reflecting the adaptation to native environment conditions; (5) Random forest model and PLS-PM confirmed that low-altitude seedlings tended to adjust leaf morphology to regulate leaf nutrients and photosynthetic physiological functions, while high-altitude seedlings regulated physiological functions by adjusting leaf nutrient changes. Seedlings from different provenance altitudes had differential adaptation pathways and regulatory strategies in response to altitude changes.

## 1 Introduction

Climate change along mountain elevations serves as a concrete manifestation of global climate change at the local scale, reflecting a microcosm of global climate change (Giménez-Benavides et al., 2018; Pepin et al., 2022). Environmental changes are particularly significant in mountainous areas (Ohmura, 2012), among which, temperature changes not only directly affect the heat balance of the microenvironment but also indirectly regulate the spatiotemporal distribution of other ecological factors such as water and light (Hansen et al., 2006; Pepin et al., 2022). Elevation, as an important variable in mountain environments, has a significant impact on the physiological state and functional morphology of seedlings (Gworek et al., 2007; Wang Q. et al., 2017). Furthermore, differences in temperature, humidity, air pressure, and radiation conditions among different elevation regions can directly act on seedlings, influencing their growth rate and physiological characteristics (Körner, 2007). Simultaneously, elevational conditions change also affect the nutrient absorption and physiological regulation mechanisms of seedlings by altering vegetation community structure or soil properties (Oldfather et al., 2016). Plants in their native environment gradually form physiological and metabolic characteristics and morphological construction patterns compatible with the local environment; these adaptive changes are the results of long-term responses to conditions such as light and temperature in the native habitat. When seedlings are transferred to different elevations, their growing environmental conditions change accordingly, and they often adjust their own resource utilization strategies and organ structural characteristics to enhance their adaptive capacity in unfamiliar environments (Ma et al., 2020). These differences in adaptive characteristics caused by different elevation backgrounds are the results of plants adapting to specific habitats and are important bases for revealing their ecological adaptation strategies (Oleksyn et al., 1998; Wang et al., 2022; Ye et al., 2020). Specifically, the adaptation mechanisms formed in the native elevation environment may enable seedlings to exhibit relatively stable performance in morphological characteristics such as photosynthetic systems, plant height, and crown width to adapt to native conditions like light and temperature. When seedlings are transplanted to new elevation regions, to cope with unfamiliar environmental factors such as temperature, humidity, and soil nutrients, their nutrient reserve strategies and structural parameters such as leaf thickness and stomatal density will undergo more obvious adjustments to improve their survival ability in the new environment (Huang et al., 2022; Ivanov et al., 2022; Zhang et al., 2023). Therefore, the change of elevational conditions is not only a comprehensive manifestation of environmental heterogeneity, but also a key entry point for analyzing plant physiological adaptability and ecological response mechanisms. We thus propose Hypothesis 1): Elevation regulates the physiological functions, nutrients, and morphology of seedlings, where native elevation mainly affects photosynthetic and morphological traits, while transplantation elevation significantly changes nutrient reserves and leaf structural parameters.

Current studies mainly focus on differences in genetic background, stress resistance, and growth and development of seedlings from different elevation provenances, revealing their specific physiological regulatory strategies under stresses such as drought, high temperature, or low temperature (Espinoza et al., 2021). Meanwhile, studies on physiological adaptation to elevation mainly concentrate on the effects of environmental factors such as temperature and humidity on physiological traits of seedlings, such as photosynthetic rate, water use efficiency, and respiration (Guo et al., 2016; Oleksyn et al., 2020; Rahman et al., 2020). However, most current studies rely on indoor control experiments, neglecting the complex effects and interactions among factors in natural mountain ecosystems, which to some extent limits the accuracy and universality of the results (Benomar et al., 2015). Additionally, existing studies pay insufficient attention to provenance differences, overlooking their impact on physiological adaptability. More critically, the synergistic regulatory mechanisms between the adaptation of seedlings in the native environment and phenotypic integration after transplantation have not been fully analyzed. This affects the existing understanding of plant adaptation to cross-elevation environments, potentially hindering the comprehension of mountain forest cultivation under the background of climate change. For regional ecosystems, it further leads to a lack of ecological restoration constraints and migration risk assessment (Bhatt and Ram, 2007). Therefore, the issues we need to address are: in which aspects does the native elevation affect the adaptability of seedlings, and what physiological changes occur after transplantation of provenances? Existing studies have shown that in the process of environmental changes, high-elevation plants often exhibit a conservative carbon metabolism strategy due to their adaptation to the native environment, and changes in soil conditions are an important part of this native environment that should also be acknowledged, whereas low-elevation plants respond to environmental fluctuations through stronger plasticity (Wu et al., 2022; Zhu et al., 2022). This differentiation in metabolic strategies and plasticity due to elevation differences is likely derived from the long-term influence of the native elevation environment, that is, light and temperature conditions at different elevations continuously act on the physiological systems of seedlings, enabling them to gradually form characteristics matching the native environment in energy conversion modes and environmental response flexibility (Gong et al., 2023; Zhu et al., 2024). When seedlings face environmental changes caused by elevation migration, these basic characteristics shaped by the native environment will further affect the adjustment range and direction of their physiological state (Reich et al., 1996a; Zhang et al., 2020). Based on this, we propose Hypothesis 2): The influence of native elevational environmental changes on seedlings focuses on photosynthetic characteristics and metabolic traits. Among them, low-elevation seedling sources show stronger environmental dynamic plasticity, while high-elevation seedling sources, due to adaptation to their native environment have limited plasticity.

*Picea asperata* is an endemic tree species, mainly distributed in North China. It has important ecological value in soil and water conservation and maintaining the stability of mountain ecosystems, and is also an important timber tree species with high economic value (Zhang J. et al., 2019). Furthermore, the current understanding of how elevation affects seedling growth and its regulatory mechanisms is not comprehensive; therefore, a deeper understanding of seedling changes and regulatory mechanisms under elevation regulation is needed (Alexander et al., 2018; Halbritter et al., 2018). In view of the above status and problems, this study takes *Picea asperata* seedlings as the research object and designs a mountain transplantation experiment based on different elevation provenances to explore their physiological adaptation mechanisms to cross-elevation environments. The selected provenances, due to differences in their native elevation environment, have formed significantly different ecotypes through long-term adaptation. By transplanting them to different elevation gradients, we systematically measured the physiological function traits (such as photosynthetic parameters, transpiration rate), morphological traits (such as branch, leaf), and nutrient traits (such as carbon and nitrogen content, carbon storage substance content) of the seedlings. By analyzing the dynamic changes of these traits, we analyzed the adaptive characteristics of different provenances after cross-elevation transplantation, and further revealed the intrinsic coupling relationships and regulatory pathways among photosynthetic physiological functions, morphology, and nutrients. Existing studies have shown that in the context of the change of elevational conditions in mountain ecosystems, low-elevation plants are more likely to adjust their morphology to regulate physiological functions, while high-elevation plants tend to rely more on regulating nutrient storage to maintain physiological functions (Tang et al., 2021; Xu et al., 2024). Combined with the theoretical basis of the native environment conditions effect of provenances mentioned above (i.e., native elevation influences seedling adaptation strategies through long-term evolutionary adaptation), we expect that *Picea asperata* seedlings from different provenances will show differential pathways in cross-elevation adaptation. Based on this, Hypothesis 3) is proposed: Low-elevation provenances mainly achieve adaptation through morphological regulation to modulate photosynthetic physiological functions, while high-elevation provenances are more influenced by the pathway of nutrient regulation of photosynthetic physiological functions.

## 2 Research methods

### 2.1 Study area and plot selection

The study site is located in Pangquangou National Nature Reserve of Guandi Mountain, Jiaocheng County, Lvliang City, Shanxi Province (37°45'~37°55'N, 111°22'~111°33'E), in the middle section of Lvliang Mountains. The altitude span is large, with the highest peak being Xiaowen Mountain at 2,831 m and the lowest altitude at 1,500 m. It belongs to the transition zone from the warm temperate zone to the mid-temperate zone, with a warm temperate monsoon continental mountain climate. The dominant soil type in the study area is mountain brown soil. The annual average temperature is 4.3°C, the relative humidity is 70%, and the annual average precipitation is about 820 mm, mostly concentrated in July-August. The forest stands in the region are neat, with various vegetation types such as natural secondary pure forests of *Larix principis-rupprechtii*, mixed forests of *Larix principis-rupprechtii and Picea asperata*, pure forests of *Pinus tabuliformis*, and broad-leaved mixed forests dominated by *Populus* and *Betula*. Understory shrubs include *Hippophae rhamnoides*, *Rosa xanthina*, etc., and herbs include *Fragaria orientalis*, *Carex tristachya*, etc.

This mountain ecosystem has significant representativeness in responding to climate change. The significant altitude difference in the region and the accompanying variation in vegetation types provide a natural ecological experimental scenario for exploring the correlation between the transplant adaptation of seedlings at different altitudes and climate change. This environment formed based on the natural altitude gradient makes the region an ideal research platform for studying seedling responses to altitude. We selected four different altitude habitats in the study area: (1600 ± 50) m, (1900 ± 50) m, (2100 ± 50) m, and (2400 ± 50) m. In each habitat, 3 forest gaps with uniform distribution, moderate distance, similar light conditions, and a size of 20 m×20 m were selected to ensure the consistency of environmental factors (temperature, precipitation, soil, etc.) and facilitate the study of the relationship between vegetation changes along the altitude gradient and environmental factors.

### 2.2 Experimental design and materials

In August 2018, 300 *Picea asperata* seedlings aged 2–3 years, with a height of (50 ± 10) cm and good growth, were selected from *Picea asperata* forests around the 1600 m and 2400 m plots (horizontal distance ≤ 200 m), which were recorded as low-altitude seedlings and high-altitude seedlings, respectively. At the end of September, the seedlings were lifted and transplanted immediately. the 300 low-altitude seedlings and 300 high-altitude seedlings were each randomly divided into 4 groups (75 seedlings per group, corresponding to 4 transplanting elevations). Each transplanting elevation included 3 replicate plots, with 25 high-altitude seedlings and 25 low-altitude seedlings planted in each plot (sourced from the 75-seedling group of each altitude type). Each plot was further divided into 2 subplots, one for each seedling source. Each seedling source was planted in 5 rows with a row spacing of 20 cm, 5 plants per row, and a plant spacing of 10 cm. The site selection of this study area took into account the representativeness of the terrain, and it includes typical plots with different slope aspects to ensure that the research results can reflect the overall situation of the region, rather than being restricted by a single slope aspect.

### 2.3 Determination methods of physiological characteristics

#### 2.3.1 Light curves of photosynthesis

In late July 2020, healthy sunlit one-year-old needle-bearing twigs from 5 randomly selected seedlings of each native altitude in each plot were used to determine the light response curve in the plot. A LI-6400 photosynthesis system with a LI-6400–22 controllable light cluster leaf chamber and a LI-6400–18 tricolor light source was used for the measurement. After placing the selected needles into the leaf chamber, they were acclimated for 20 minutes to stabilize photosynthetic parameters before initiating the light response curve measurement. The system was set to determine the plants’ light response curve under an effective photosynthetic radiation of 1200 μmol m-^2^ s-^1^, and the effective photosynthetic radiation gradient was set as 0, 50, 100, 200, 400, 600, 800, 1000, 1200, 1400, 1600 μmol m-^2^ s-^1^. To obtain a suitable and stable environment, a CO_2_ cylinder was used to provide a stable CO_2_ source of 380 ppm during determination, all measurements were carried out from 9:00 to 11:30 am, and the leaf temperature was maintained at 25°C. The light response curves were fitted using the non-rectangular hyperbola model. Net photosynthetic rate (Pn) was derived from these fitted curves, while transpiration rate (Tr), stomatal conductance (gsw), and water use efficiency (WUE) were calculated based on the measured data.

The fitting model for Pn is given by:


$$Pn=\frac{\alpha I+Pn_{\max}-\sqrt{{\left(\alpha I+Pn_{\max}\right)}^{2}-4\theta \alpha IPn_{\max}\;}}{2\theta }-Rd$$

Note: *Pn* (μmol m^−2^ s^−1^) is the net photosynthetic rate; *α* (μmol μmol^−1^) is the apparent quantum efficiency; *I* (μmol m^−2^ s^−1^) is the photosynthetic photon flux density; *Pn_max_* (μmol m^−2^ s^−1^) is the maximum net photosynthetic rate; *Rd* (μmol m^−2^ s^−1^) is the dark respiration rate.

#### 2.3.2 Determination of morphological traits

In August 2020, 5 high-altitude seedlings and 5 low-altitude seedlings were selected from each of the 4 transplant plots to determine the new branch growth and leaf morphological traits, with a total of 40 seedlings measured. A steel ruler and vernier caliper were used to measure 5 healthy new branches per seedling to determine the new branch length (BL) and basal diameter (DBA); the final values of BL and DBA for each seedling were calculated as the average of these 5 measured branches. The branch slenderness index (Slender = BL/DBA) was derived from the average BL and DBA values of each seedling. Additionally, relevant samples were collected from each of 5 high-altitude seedlings and 5 low-altitude seedlings; afterward, the samples from each individual seedling were separately divided into 3 portions, and all samples were brought back to the laboratory in an incubator. Fresh leaf samples were taken, and leaf morphological parameters were determined using the WinSEEDLE leaf analysis system. Mature needles were collected, washed with deionized water, and dried. High-resolution leaf images were obtained using a flatbed scanner, imported into the software, and the color threshold was set to identify the leaf contour. Leaf length (SL), leaf width (SW), and leaf area (PA) were measured. The leaf length-width ratio (LW = SL/SW) and specific leaf area (SLA = PA/DW) were further calculated. Since specific leaf area (SLA) measurement requires coupling with leaf dry weight (DW), the collected leaves were first subjected to a drying process: they were deactivated at 105°C for 15–30 minutes to stop enzymatic activity, then dried at 80°C to a constant weight (i.e., no further change in weight was observed in consecutive weighing). After drying, an electronic balance was used to determine the leaf dry weight (DW). During the entire measurement process, errors were controlled through repeated scanning verification to ensure data accuracy.

#### 2.3.3 Determination of nutrient traits

Several healthy needles from 5 high-altitude seedlings and 5 low-altitude seedlings were collected, mixed, divided into 3 parts, and brought back to the laboratory in an incubator. One part of the fresh leaf sample was used to determine the leaf soluble protein (Protein) content by ultraviolet absorption method (Noble, 2014). One part of the fresh leaf sample was dried in a 68°C blast drying oven; an electronic balance was used to determine leaf dry weight (DW); a total carbon analyzer [Analytik Jena (Beijing) Co., Ltd.] was used to determine leaf carbon (C) content; the Kjeldahl method was used to determine leaf Nmass content (Quirino et al., 2023); the anthrone colorimetric method was used to determine leaf soluble sugar (Sugar) and starch (Starch) content (Quentin et al., 2015). The content of non-structural carbohydrates (NSC = Sugar + Starch) was calculated.

### 2.4 Data analysis methods

The experiment was designed as a field experimental setup, with the same number of seedlings allocated to each plot. However, one year after transplantation, the survival rate of seedlings differed across different plots, and the number of surviving seedlings varied significantly. Therefore, we could not conduct data statistics as per the original experimental design, and instead used "transplanting altitude" and "provenance altitude" as the two factors for data analysis. Data were analyzed by two-way analysis of variance (ANOVA) for plot and provenance using R4.5.0 software, and multiple comparisons were performed using LSD test for traits with significant differences in ANOVA (P< 0.05); circular bar charts were used to visualize the trend differences of regenerated seedlings under different treatments; the importance and correlation of seedling morphological and nutrient traits with photosynthetic physiological functions were evaluated based on %IncMSE analysis of the random forest model; finally, PLS-PM models were constructed separately for the two groups of seedlings (low-altitude seedlings originating from 1,600 m and high-altitude seedlings originating from 2,400 m) to clarify the divergent pathways by which transplanting altitude affects their photosynthetic physiology. For each group of seedlings, the model included samples from all their transplanted elevations, with variables covering morphological traits, nutrient traits, and photosynthetic physiological indices, aiming to reveal how altitude-driven changes in traits cascade to affect photosynthetic functions within each group of seedlings. This provides data support for revealing the adaptation strategies of seedlings from different altitudes.

## 3 Data analysis and results

### 3.1 ANOVA results

We analyzed the significance of native altitude, transplantation altitude, and their interaction on *Picea asperata* seedlings. (**Table 1**).

**Table 1:** Factor effects on seedling traits (ANOVA: F & P-values).

Factor	Statistic	Protein	Sugar	NSC	C	Nmass	CNRatio	AvgPA	AvgSL	AvgSW
Native elevation	F-value	1.17	0.76	0.08	0.15	4.36	3.13	0.54	3.33	0.49
	P-value	0.29	0.39	0.78	0.70	<0.05 *	0.09	0.47	0.08	0.49
Transplantation elevation	F-value	8.99	6.48	8.23	2.74	2.75	1.64	4.24	4.18	4.68
	P-value	<0.001 ***	<0.01 **	<0.001 ***	0.06	0.06	0.20	<0.05 *	<0.05 *	<0.01 **
Native elevation× Transplantation elevation	F-value	1.61	15.10	11.09	4.47	0.74	1.88	4.35	4.03	4.53
	P-value	0.21	<0.001 ***	<0.001 ***	<0.01 **	0.53	0.15	<0.05 *	<0.05 *	<0.01 **
Factor	Statistic	LWRatio	SLA	BL	DBA	Slender	Pn	Trmmo	WUE	gsw
Native elevation	F-value	1.52	21.15	2.56	0.74	0.17	29.67	3.82	16.03	77.22
	P-value	0.23	<0.001 ***	0.12	0.40	0.68	<0.001 ***	0.06	<0.001 ***	<0.001 ***
Transplantation elevation	F-value	1.13	12.88	2.43	0.72	0.20	3.14	21.67	1.70	0.86
	P-value	0.35	<0.001 ***	0.08	0.55	0.89	<0.05 *	<0.001 ***	0.19	0.47
Native elevation× Transplantation elevation	F-value	0.32	7.27	0.12	0.04	0.25	6.62	10.09	2.39	2.91
	P-value	0.81	<0.001 ***	0.95	0.99	0.86	<0.01 **	<0.001 ***	0.09	<0.05 *

In the table, Protein, leaf protein content; Sugar, mass-based leaf soluble sugar content; NSC, leaf non-structural carbohydrate content; C, leaf carbon content; Nmass, mass-based leaf nitrogen content; CNRatio, C/N ratio; AvgPA, average projected leaf area; AvgSL, average leaf length; AvgSW, average leaf width; LWRatio, leaf length-width ratio; SLA, specific leaf area; BL, new branch length; DBA, branch diameter; Slender, branch slenderness index; Pn, net photosynthetic rate; Trmmo, transpiration rate; WUE, water use efficiency; gsw, stomatal conductance. * in the table indicates statistical significance, where *P< 0.05; **P< 0.01; ***P< 0.001.

The interaction between native and transplantation altitudes had highly significant effects on nutrient traits (Sugar, NSC, C) and significant effects on physiological traits (Pn, Trmmo, gsw) and morphological traits (AvgPA, AvgSL, AvgSW, SLA). For morphological traits with no significant interaction (LWRatio, BL, DBA, Slender), their individual effects were analyzed separately, with neither native nor transplantation altitude exerting significant effects on these four traits. In addition, native altitude exerted extremely significant effects on physiological traits (Pn, WUE, gsw) and morphological indicator (SLA) (P< 0.001), and a significant effect on nutrient trait Nmass (P< 0.05); transplantation altitude significantly affected nutrient traits (NSC, Sugar, Protein) (P< 0.01).

### 3.2 Changes in physiological, morphological, and metabolic parameters of *Picea asperata* from different native altitudes

#### 3.2.1 Changes in photosynthetic physiological functions

We found that the physiological traits Pn, Trmmo, and gsw of low-altitude seedlings showed a trend of first increasing and then decreasing with the increase of transplantation altitude, reaching a peak when transplanted at 1900 m. WUE showed a trend of first decreasing and then increasing with the increase of transplantation altitude, with the valley value also appearing at the transplantation altitude of 1900 m. However, all physiological traits of high-altitude seedlings showed a continuous downward trend with the decrease of transplantation altitude.

**Figure 1:**
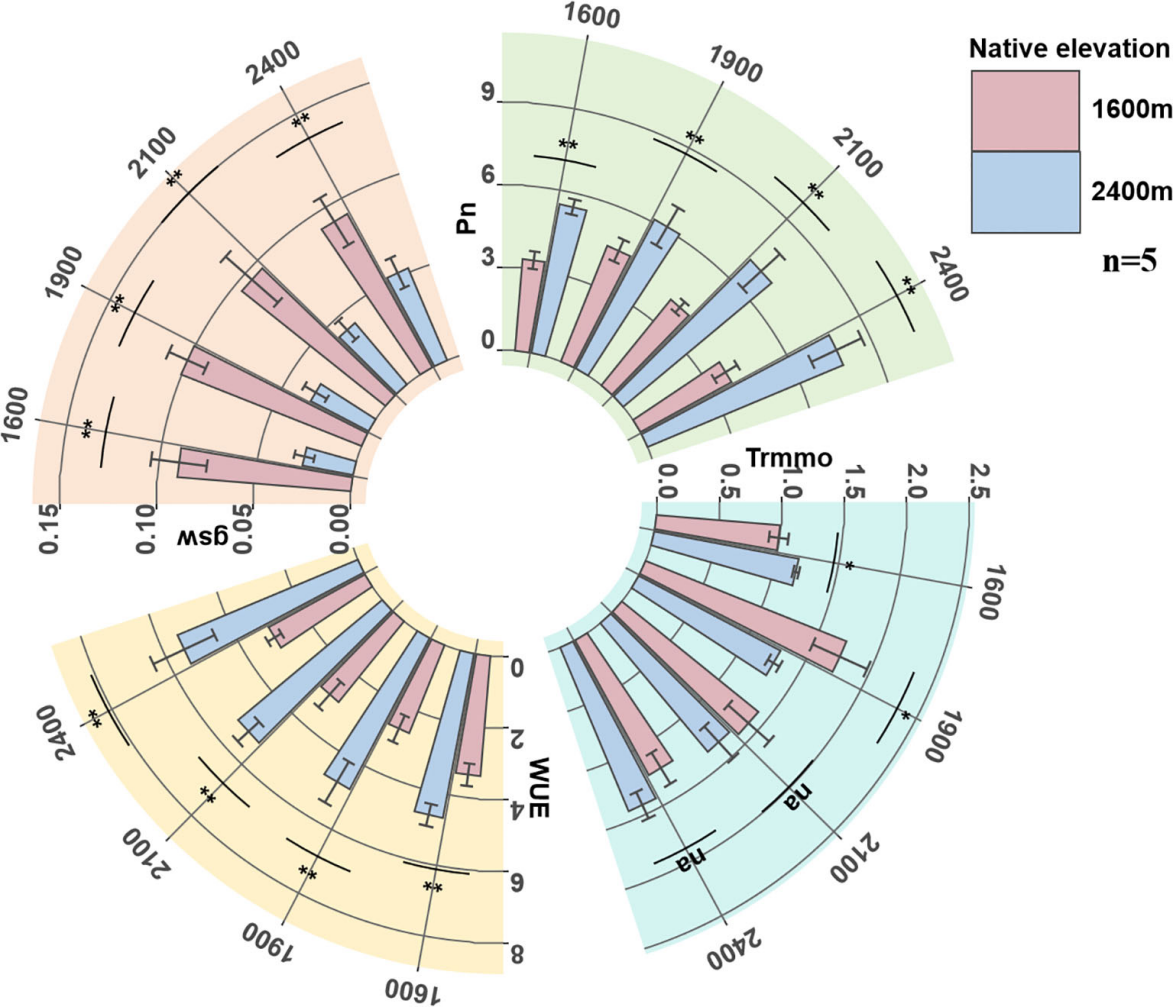
The changes of seedling Pn, Trmmo, WUE and gsw. Pn, net photosynthetic rate (μmol CO_2_ m-^2^ s-^1^); Trmmo, transpiration rate (mmol H_2_O m-^2^ s-^1^); WUE, water use efficiency (μmol CO_2_ mmol-^1^ H_2_O); gsw, stomatal conductance (mol H_2_O m-^2^ s-^1^).

In comparison, the Pn and WUE of high-altitude seedlings at their native altitude were significantly higher than those of low-altitude seedlings at any transplantation altitude, but their gsw was significantly lower than the latter. Low-altitude seedlings showed peak responses of multiple physiological traits at medium transplantation altitude, while high-altitude seedlings did not show similar adaptive peaks (**Figure 1**).

#### 3.2.2 Changes in morphological traits

With the increase of transplantation altitude, the leaf structure (AvgPA, AvgSL, AvgSW) and SLA of low-altitude seedlings reached peaks when transplanted at 1900 m, showing a trend of first increasing and then decreasing, while the new branches (DBA, BL) and branch metric ratio (Slender) showed a continuous upward trend with the increase of transplantation altitude. With the decrease of transplantation altitude, the leaf structure, new branches, SLA, and LWRatio of high-altitude seedlings all showed a continuous downward trend, while Slender continued to increase, and the branches became thinner.

**Figure 2:**
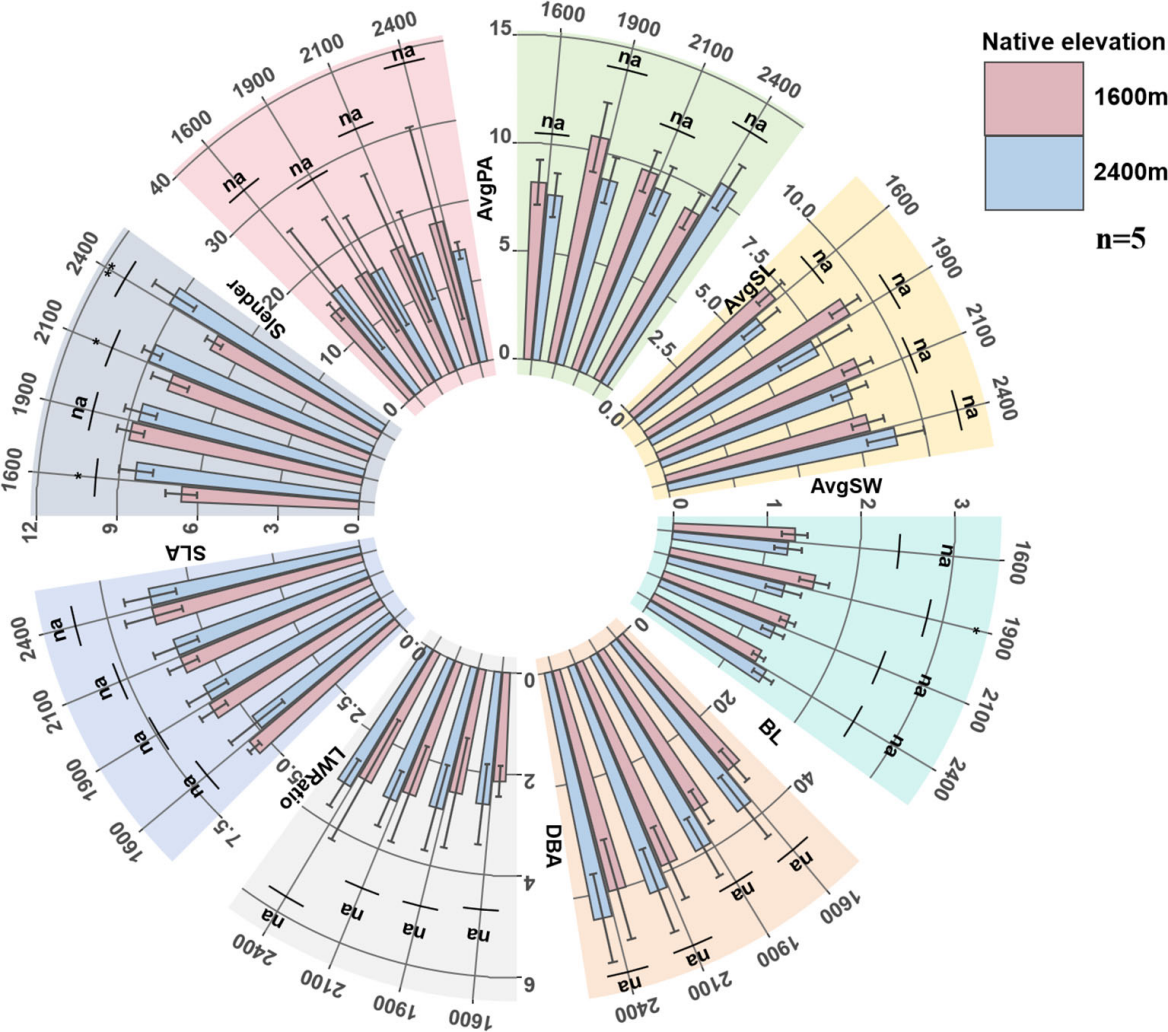
The changes of seedling AvgPA, AvgSL, AvgSW, BL, DBA, LWRatio, SLA and Slender. AvgPA, average projected leaf area (cm^2^); AvgSL, average leaf length (cm); AvgSW, average leaf width (cm), LWRatio: leaf length-width ratio; SLA, specific leaf area (cm^2^g-^1^); BL, new branch length (cm); DBA, branch diameter (cm); Slender, branch slenderness index.

From the above trait variation trends, low-altitude seedlings only achieved leaf morphological advantages at medium transplantation altitude, and their new branches were always shorter than those of high-altitude seedlings, though the differences in branch length were not statistically significant. The new branches of high-altitude seedlings in their native habitat were longer than those of low-altitude seedlings at any transplantation altitude (**Figure 2**).

#### 3.2.3 Changes in nutrient traits

With the increase of transplantation altitude, the carbon storage substances (NSC, Sugar) of low-altitude seedlings showed a trend of first increasing and then decreasing, with the peak appearing at the transplantation altitude of 1900 m. Protein continued to decrease, and carbon content synchronously reached the peak at 1900 m, while nitrogen content continued to decrease, resulting in a continuous increase of CNRatio with transplantation altitude. With the decrease of transplantation altitude, the carbon storage substances and carbon content of high-altitude seedlings showed a continuous downward trend, while Protein and nitrogen content continued to increase after decreasing at 2100 m, resulting in a synchronous decrease of CNRatio with altitude after increasing at 2100 m.

**Figure 3:**
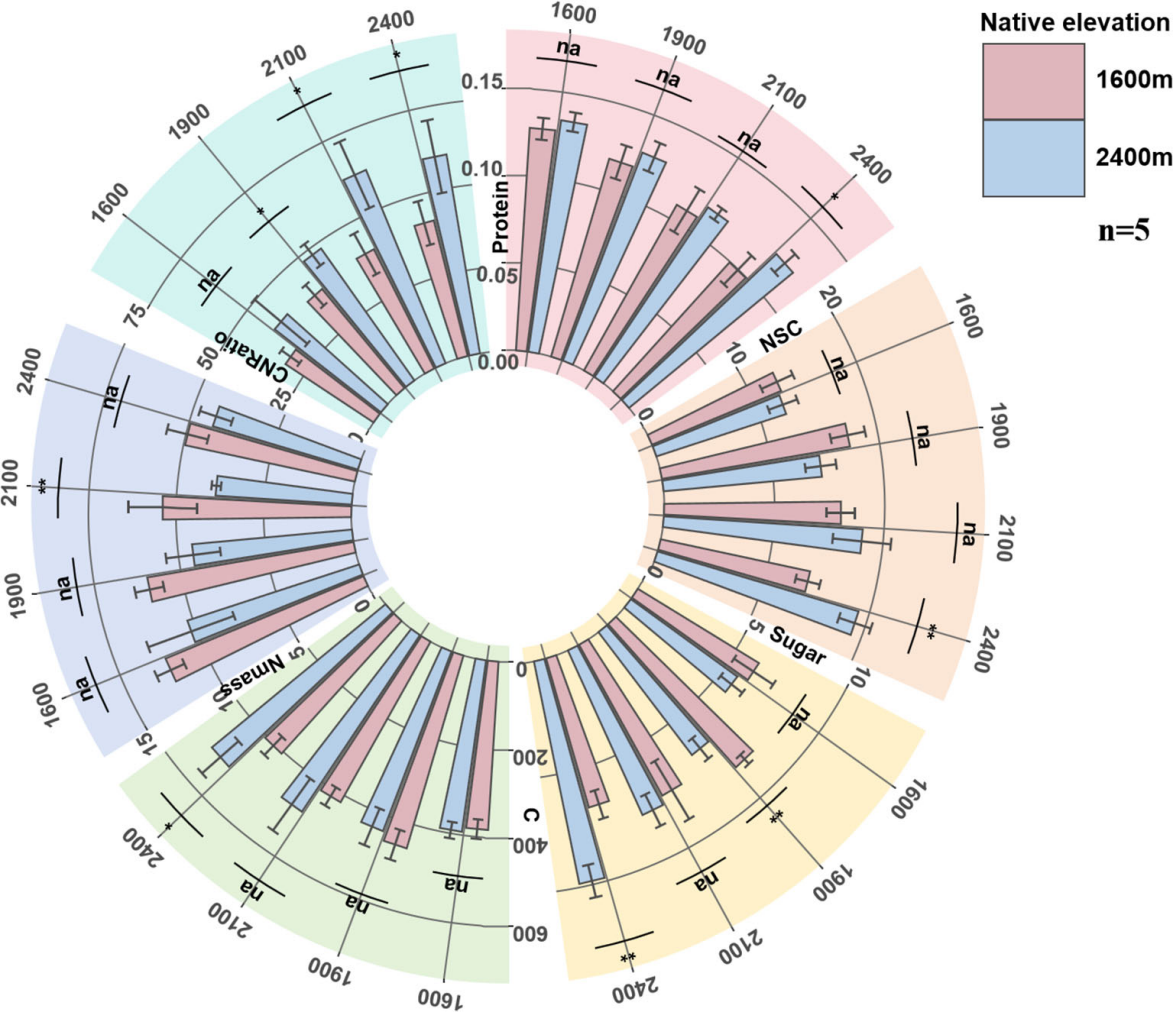
The changes of seedling Protein, NSC, Sugar, C, Nmass, CNRatio. Protein, leaf protein content (mg g-^1^); Sugar, mass-based leaf soluble sugar content (mg g-^1^); NSC, leaf non-structural carbohydrate content (mg g-^1^); C, leaf carbon content (g kg-^1^); Nmass, mass-based leaf nitrogen content (g kg-^1^); CNRatio, C/N ratio.

In comparison, when high-altitude seedlings grow in their native altitude environment, their carbon content and CNRatio are higher than those measured in low-altitude seedlings when the latter grow in any transplanted altitude environment, while the nitrogen content of low-altitude seedlings in their native habitat was higher. The carbon storage substances and carbon content of low-altitude seedlings reached peaks when transplanted at 1900 m, which were synchronous with their physiological functions and leaf morphology (**Figure 3**).

### 3.3 Relationship between physiological functions and morphological and nutrient traits of *Picea asperata*

The random forest model revealed the difference in contributions of morphological and nutrient traits to photosynthetic physiological functions through variable importance ranking. Based on the %IncMSE analysis from the random forest model, which highlighted physiological traits (Pn, Trmmo), these key traits are highly regulated by native/transplant altitudes and exhibit stronger altitudinal responses than structural or nutrient traits.

#### 3.3.1 Importance evaluation of morphological and nutrient traits to Pn

**Figure 4:**
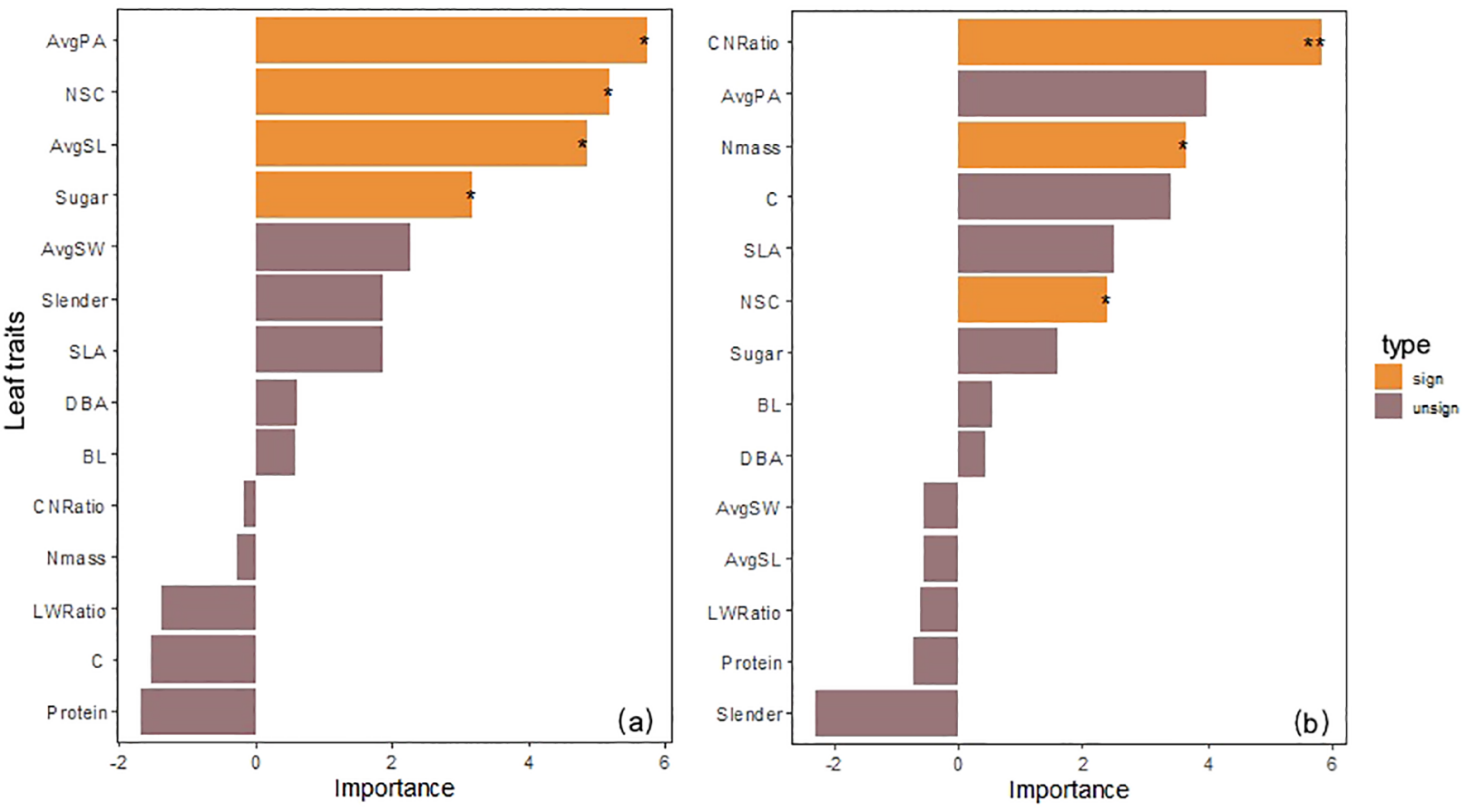
**(a)** Evaluation of the importance of morphological and nutrient traits to net photosynthetic rate of seedlings from the 1600 m provenance. **(b)** Evaluation of the importance of morphological and nutrient traits to net photosynthetic rate of seedlings from the 2400 m provenance.

Random forest analysis results showed that among 14 possible predictors, 4 factors significantly explained the changes in Pn of low-altitude seedlings: AvgPA (%IncMSE = 5.72, P< 0.05), AvgSL (%IncMSE = 4.84, P< 0.05) in leaf morphological structure, and NSC (%IncMSE = 5.19, P< 0.05), Sugar (%IncMSE = 3.17, P< 0.05) in carbon storage substances; while the significant influencing factors of high-altitude seedlings turned to CNRatio (%IncMSE = 5.82, P< 0.01), Nmass (%IncMSE = 3.66, P< 0.05), and NSC (%IncMSE = 2.41, P< 0.05) in nutrient traits. It is worth noting that CNRatio changed from a non-significant factor in low-altitude seedlings (%IncMSE = -0.16) to the strongest influencing factor in high-altitude seedlings, and NSC was a common significant influencing factor for both low-altitude and high-altitude seedlings (**Figure 4**).

#### 3.3.2 Importance evaluation of morphological and nutrient traits to Trmmo

**Figure 5:**
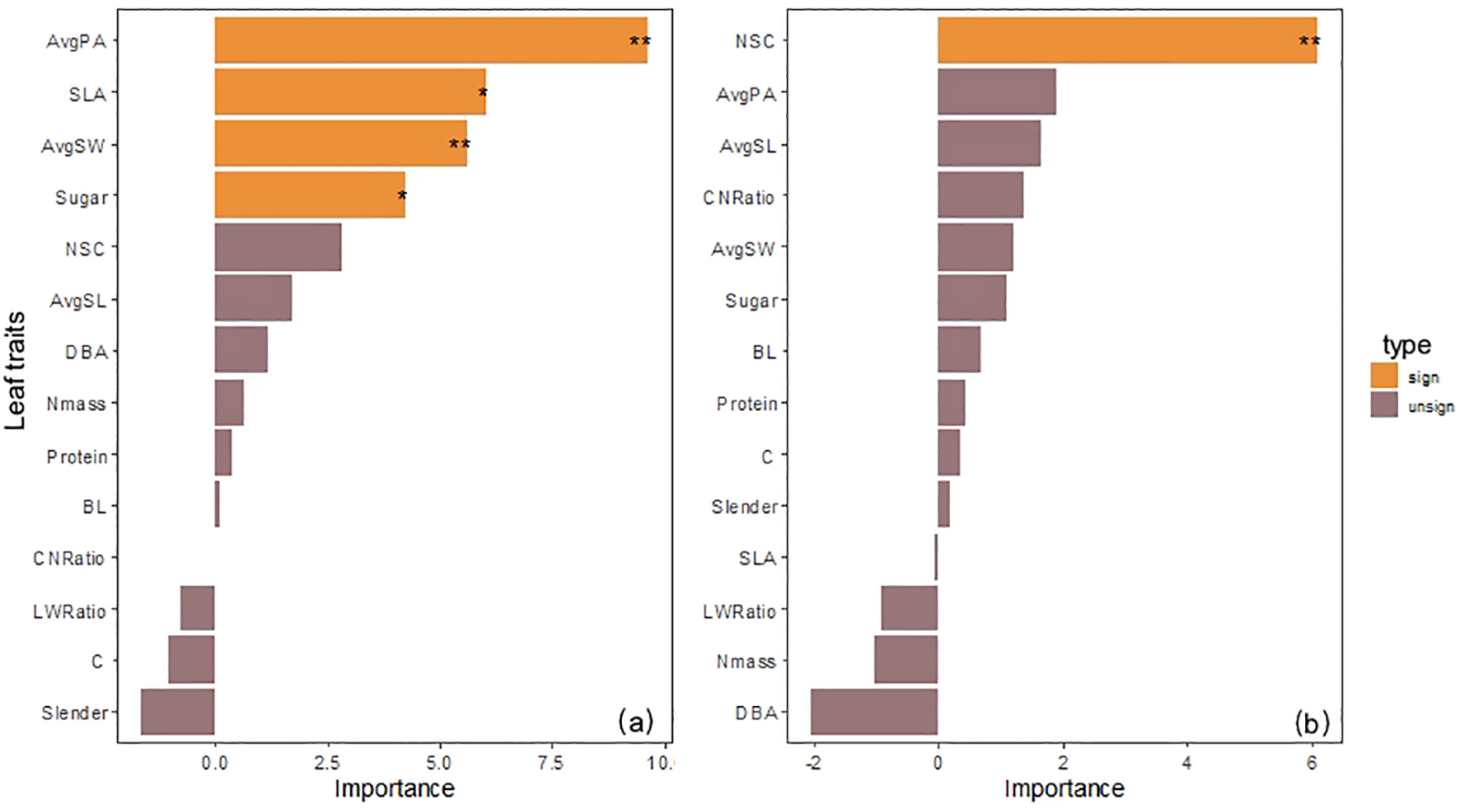
**(a)** Evaluation of the importance of morphological and nutrient traits to transpiration rate of seedlings from the 1600 m provenance. **(b)** evaluation of the importance of morphological and nutrient traits to transpiration rate of seedlings from the 2400 m provenance.

Random forest analysis results showed that among 14 possible predictors, 4 factors significantly explained the changes in Trmmo of low-altitude seedlings: AvgPA (%IncMSE = 9.60, P< 0.01), SLA (%IncMSE = 6.03, P< 0.05), AvgSW (%IncMSE = 5.60, P< 0.01) in leaf morphological structure, and Sugar (%IncMSE = 4.23, P< 0.05) in nutrient traits; while the significant influencing factor of high-altitude seedlings turned to NSC (%IncMSE = 6.08, P< 0.01) in carbon storage substances. It is worth noting that AvgPA, the strongest factor affecting low-altitude seedlings (%IncMSE = 9.60), turned to a non-significant factor at high altitude (%IncMSE = 1.89, P = 0.16), and DBA turned from (%IncMSE = 1.16) to (%IncMSE = -2.04). NSC was the core influencing factor for high-altitude seedlings (**Figure 5**).

### 3.4 Effects of transplantation altitude on photosynthetic physiological functions of *Picea asperata*

**Figure 6:**
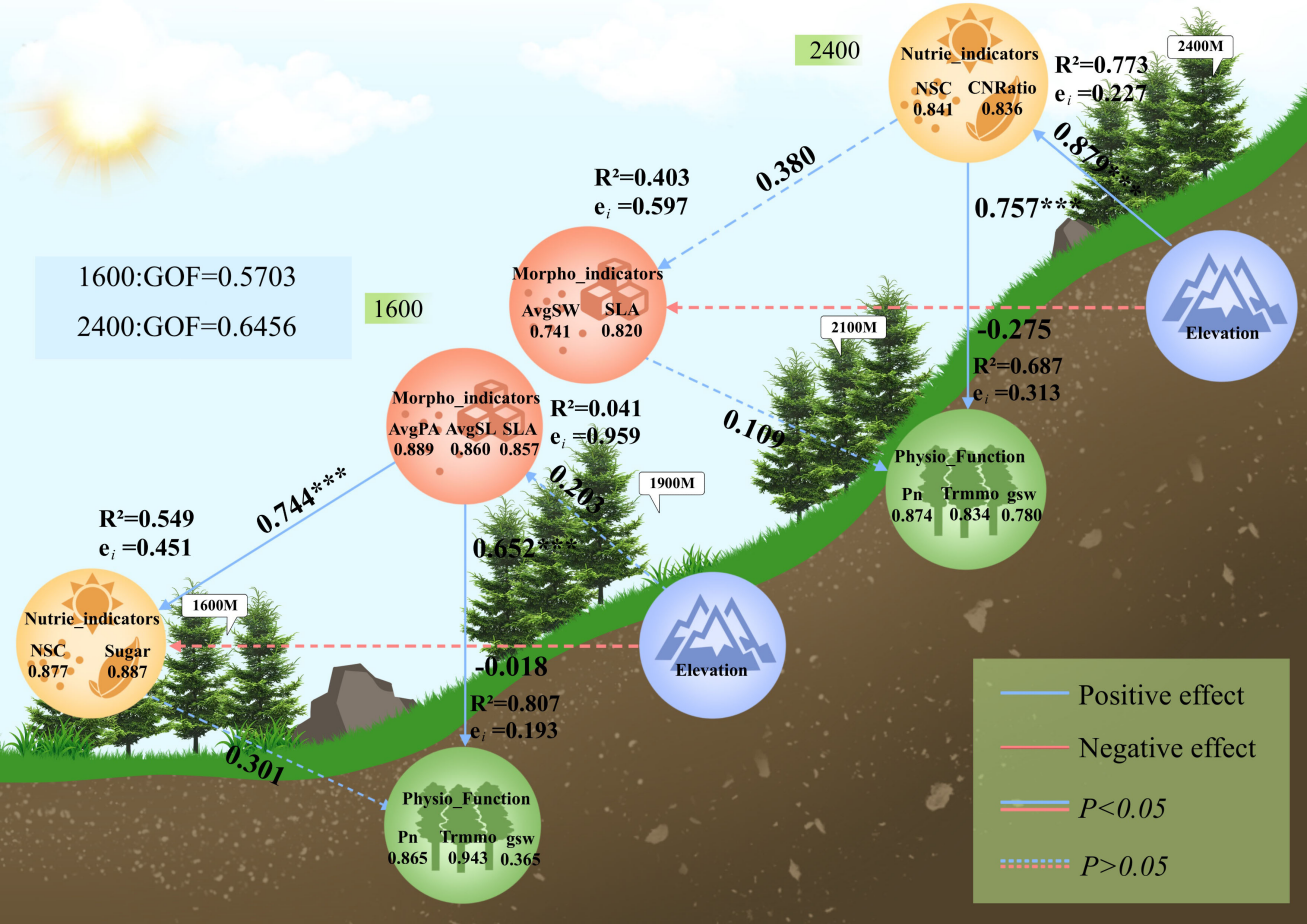
Upper one, effects of transplanting altitude on photosynthetic physiological functions of high-altitude seedlings (2400m), lower one, effects of transplanting altitude on photosynthetic physiological functions of low-altitude seedlings (1600m). In the figure, Sugar, mass-based leaf nitrogen content; NSC, leaf non-structural carbohydrate content; CNRatio, C/N ratio; AvgPA, average projected leaf area; AvgSL, average leaf length; AvgSW, average leaf width; SLA, specific leaf area; Pn, net photosynthetic rate; Trmmo, transpiration rate; gsw, stomatal conductance.

According to the results of the final PLS-PM model, the possible relationships between morphological traits, nutrient traits, and physiological functions under altitude environmental changes were illustrated. There are two models in **Figure 6**, and their coefficients of determination (R²) are distinguished by seedling type: the R² of the model for low-altitude seedlings in **Figure 6** was 0.807, indicating that this model explained 80.7% of the variance in the data, with a strong ability to explain physiological functions; the R² of the model for high-altitude seedlings in **Figure 6** was 0.687, indicating that this model explained 68.7% of the variance in the data, with a strong ability to explain physiological functions.

#### 3.4.1 Effects of transplanting altitude on seedlings from 1600 meters

In the external module of morphological traits, the results showed that AvgPA was the most important predictor of morphological traits (Loading = 0.89), followed by AvgSL (Loading = 0.86) and SLA (Loading = 0.86). In the nutrient indicator module, the predictive importance of NSC (Loading = 0.88) and Sugar (Loading = 0.89) was comparable. In the physiological function module, Trmmo was the strongest predictor (Loading = 0.94), followed by Pn (Loading = 0.87); while the predictive importance of gsw was low (Loading = 0.36), but we consider it still has a certain explanatory power in this model and thus retained it.

In the internal model, the direct effect of transplantation altitude on morphological traits was not significant (β = 0.203, t = 0.878, P = 0.392), but morphological traits had a strong positive predictive effect on nutrient traits (β = 0.744, t = 4.47, P< 0.001), and also had a significant direct effect on physiological functions (β = 0.652, t = 4.11, P< 0.001). However, the direct effect of nutrients on physiological functions was not significant (β = 0.301, t = 1.90, P = 0.075).

#### 3.4.2 Effects of transplanting altitude on seedlings from 2400 meters

In the external module of nutrient traits, the results showed that the predictive importance of NSC (Loading = 0.841) and C/N ratio (Loading = 0.836) was comparable. In the morphological indicator module, SLA was the strongest predictor (Loading = 0.820), followed by AvgSW (Loading = 0.741). The key traits in the physiological function module were Pn (Loading = 0.874), Trmmo (Loading = 0.834), and gsw (0.780), all with strong importance prediction.

In the internal model, transplantation altitude had a strong direct positive effect on nutrient traits (β = 0.879, t = 7.83, P< 0.001), and the direct effect of nutrient traits on morphology did not reach a significant level (β = 0.380, t = 0.965, P = 0.348), but had an extremely significant effect on physiological functions (β = 0.757, t = 4.37, P< 0.001). The direct effect of morphological traits on physiological functions was not significant (β = 0.109, t = 0.632, P = 0.536).

## 4 Discussion

### 4.1 Factors regulating *Picea asperata* characteristics

We consider that the environmental adaptation of *Picea asperata* to its native altitude primarily involves the regulation of physiological traits (Pn, WUE, and gsw) and exerts a significant effect on SLA and nitrogen. However, its regulatory role in leaf morphological structure and carbon storage is relatively limited, failing to substantially induce changes in these two traits (Thomas, 2011). The regulatory ability of the transplantation altitude seems stronger. During the regulation by transplantation altitude, seedlings not only change leaf structure but also carbon storage (Bauk et al., 2015; He et al., 2022). However, the regulatory intensity of transplantation altitude is not sufficient to completely offset the local adaptation to the native environment (Midolo and Wellstein, 2020). The interaction between native altitude and transplantation altitude indicates that carbon storage and physiological traits (Pn, Trmmo, and gsw) are significantly affected by both, and this interaction also affects leaf structure. Existing studies have confirmed that the interaction between native altitude and transplantation altitude affects the physiological characteristics of plants: after high-altitude seedlings are transplanted to low altitude, their original carbon metabolism strategy will still be retained, while the degree of leaf morphological change in low-altitude seedlings when transplanted to a high-altitude environment is also related to their original growth environment (Golob et al., 2022; Xu et al., 2024). Meanwhile, in response to the interaction of the two, the growth strategy and adaptation path of seedlings will also change, which will be analyzed in the subsequent discussion (Ensslin et al., 2018; Ghalambor et al., 2007; Lajoie and Vellend, 2018). Therefore, the regulation of photosynthetic and morphological traits by native altitude and the influence of transplantation altitude on nutrients and leaf structure verify our Hypothesis 1). However, the influence of the interaction between native and transplantation altitude on carbon storage, physiological, and leaf morphological traits goes beyond the presupposition of Hypothesis 1), which only considers the influence of a single altitude.

### 4.2 Effects of transplantation altitude on *Picea asperata*

#### 4.2.1 Effects of transplantation altitude on photosynthetic physiological functions of *Picea asperata* from different native altitudes

Phenotypic plasticity and local adaptation are two strategies for organisms to cope with environmental changes. The same species can have both phenotypic plasticity and local adaptation characteristics, and the relative importance of the two in adapting to environmental changes is related to specific traits, ecological factors, etc (Chevin and Hoffmann, 2017; Garzón et al., 2019; Via et al., 1995).

We consider that medium altitudes may be low-altitude seedlings’ optimal growth environment, free from the high-temperature stress of low altitudes or the low-temperature limitation of high altitudes (Rahman et al., 2020; Wu et al., 2023). WUE first decreased and then increased with altitude, with the minimum value appearing at 1900 m. Because at this altitude, gsw, Trmmo, and Pn increased synchronously, which means that although the increase in gsw led to increased water consumption, plants could still effectively use water due to the enhancement of photosynthesis, thus achieving growth capacity improvement (Dong et al., 2023; Vats and Kumar, 2006). However, with the further increase in altitude, the enhancement of stomatal limitation promoted the recovery of WUE (Bertolino et al., 2019; Caldera et al., 2017). Therefore, low-altitude seedlings have stronger physiological functions at medium altitude, and at high altitude, they reduce Trmmo through stomatal limitation. Although Pn decreases accordingly, WUE is improved by reducing water loss, which fully reflects the dynamic physiological plasticity of low-altitude seedlings in different environments (Lombardozzi et al., 2012; Wang H. et al., 2017). The photosynthetic physiological functions of high-altitude seedlings showed a continuous downward trend with the decrease in transplantation altitude, reflecting their limited ability to acclimate to the transplantation environment (García-Plazaola et al., 2015). However, high-altitude seedlings gsw was significantly lower than that of low-altitude seedlings, indicating that they improved WUE by reducing stomatal opening to achieve higher Pn and promote growth, but this weakened gas exchange capacity, showing adaptive characteristics to the native environment (Zhai et al., 2024; Zhang et al., 2015). Verifying that the environmental conditioning by the native altitude focuses on the photosynthetic characteristics of seedlings (as stated in Hypothesis 2).

#### 4.2.2 Effects of transplantation altitude on morphological traits of *Picea asperata* from different native altitudes

Low-altitude seedlings achieved morphological optimization at medium altitude, while high-altitude seedlings showed insufficient acclimatory capacity to transplantation altitude changes (Hultine and Marshall, 2000; Scheepens et al., 2010).

For low-altitude seedlings, their leaf structure and SLA reached peaks at 1900 m. This aligns with their photosynthetic performance, as enhanced leaf morphology (e.g., higher SLA) likely improved light capture and gas exchange efficiency, supporting optimal photosynthesis at medium altitude (Fan et al., 2022; Peppe et al., 2011). However, beyond 1900 m, the synchronous decline in leaf traits and photosynthetic function indicates that the growth of low-altitude seedlings is limited (Barrera et al., 2000; Liu et al., 2016). With the decrease in transplantation altitude, various leaf morphological traits of high-altitude seedlings continued to decrease, indicating that their capacity for acclimation to the low-altitude environment is inferior to their adaptation to the native altitude (Reich et al., 1996b; Zhang et al., 2021). The research conclusion of Reich et al. shows that the changes in leaf traits among different plants are closely related to ecosystem attributes (Reich et al., 1997). We consider that the needles of high-altitude seedlings may be adapted to the native altitude environment, and leaf growth is better in the native environment when the latter is not limiting, which in turn supports high metabolic activity, consistent with the research of Korner et al (Korner, 1989). However, in the low-altitude environment, they may not effectively cope with higher temperature and light, leading to the degradation of leaf morphological structure (Wang et al., 2025). The new branches of low-altitude seedlings were always shorter than those of high-altitude seedlings in their native environment at all transplantation altitudes, indicating that low-altitude seedlings may prioritize leaf optimization during growth, while branch growth is relatively lagging (Premoli et al., 2007). According to the leaf economic spectrum theory: species tend to reduce investment in structural defense in exchange for faster nutrient and biomass return rates (Wright et al., 2004). Thus, the phenomenon that low-altitude seedlings give priority to leaf development to improve photosynthetic capacity indicates that low-altitude seedlings show the "quick investment and return" strategy described in the leaf economic spectrum theory (Zhong et al., 2022). High-altitude seedlings, though showing strong new branch growth in their native environment, degrade in parallel with photosynthetic decline at low altitudes. This indicates insufficient overall adaptability of high-altitude seedlings to low-altitude conditions (Fan et al., 2019). We consider that the differences in seedling morphological growth strategies are the results of native environment conditions (Luo et al., 2006). Which is consistent with Hypothesis 2) that native environment conditions lead to plasticity differentiation of seedlings, but expands the research scope from photosynthetic characteristics and metabolic traits to morphological growth strategies, which is a supplement to the hypothesis.

#### 4.2.3 Effects of transplantation altitude on nutrient traits of *Picea asperata* from different native altitudes

Low-altitude seedlings showed better carbon accumulation capacity at medium altitude, but this capacity was limited at high altitude, high-altitude seedlings showed increased needle nitrogen content at low altitude, but their overall resource accumulation capacity decreased (Wang et al., 2025).

Low-altitude seedlings’ carbon storage peaked at 1900 m during transplantation. This phenomenon indicates that synchronous improvements in photosynthesis and leaf morphology boosted carbon uptake and storage at this altitude (Wang et al., 2024). Furthermore, with the further increase in altitude, environmental changes begin to have a negative impact on their growth, leading to the limitation of carbon storage capacity and a subsequent downward trend (Jiang et al., 2024). In contrast, the carbon storage substances and carbon content of high-altitude seedlings continued to decrease with the decrease in transplantation altitude (Paschoa et al., 2024; Rahman et al., 2020), probably due to their adaptation to low temperatures and high light in their native environment (Xing et al., 2024). The nitrogen content of low-altitude seedlings was higher in their native habitat, but with the increase in transplantation altitude, the nitrogen content continued to decrease, leading to an increase in C/N ratio (Bin et al., 2022; Zhu et al., 2024). This pattern may partly reflect the gradient in soil nitrogen availability, and it also indicates that low-altitude seedlings give priority to carbon accumulation and relatively reduce nitrogen absorption at high altitude. Poorter concluded through meta-analysis that "the ability of plants to adjust allocation is usually not as good as the ability to change organ morphology" (Poorter et al., 2012). Thus, we consider that this strategy may aim to improve tolerance to high altitude and save resources by reducing nitrogen utilization (Guo et al., 2025). The nitrogen content of high-altitude seedlings showed a fluctuating trend of decreasing first and then increasing with the decrease in transplantation altitude, reflecting their better ability to utilize nitrogen resources at low altitude (Liu et al., 2024). Since soil organic carbon is the most important driving factor for the variation of nitrogen components along an altitude gradient, we consider that this fluctuation may be caused by differences in soil organic carbon at different altitudes (Meng et al., 2024; Zhu et al., 2025). By comparison, the carbon content of high-altitude seedlings at their native altitude was higher than that of low-altitude seedlings at any transplantation altitude, indicating that high-altitude seedlings exhibit stronger carbon accumulation capacity in their suitable habitat (Pan et al., 2023; Wang et al., 2022). This indicates that the native adaptation background of seedlings affects the altitude adaptation performance of seedlings (Zhang et al., 2023), but the effectiveness of plasticity and adaptability is limited by the altitude environment. When exceeding the strategy threshold, both show strategy failure. This finding is consistent with Hypothesis 2) that native altitude influence on seedlings focuses on metabolic traits, that native altitude influence affects the adaptation differentiation of provenances, and it supplements the constraint that the effectiveness of native altitude influence has an altitude environment threshold, which was not considered in Hypothesis 2).

### 4.3 Are physiological functions more affected by morphology or nutrients?

According to the random forest results, the main influencing factors for Pn changes in seedlings from different native altitudes have changed. The Pn changes of low-altitude seedlings show a significant positive correlation with leaf morphology and carbon storage substances, indicating that they affect Pn changes in the low-altitude seedlings by adjusting leaf light-capturing structure and carbon storage substance content (Wallace and Dunn, 1980; Xu et al., 2023). However, high-altitude seedlings are more affected by nutrients. The C/N ratio has a negative predictive effect for low-altitude seedlings, while for high-altitude seedlings it becomes the most important factor that, together with Nmass and NSC, significantly affects photosynthesis. Studies have shown that this is caused by resource limitation in high-altitude environments, leading to the Pn of seedlings being more affected by nitrogen and carbon storage substances (Hou et al., 2024; Klipel et al., 2023; Wang et al., 2023). NSC is a common significant influencing factor for seedlings from both altitudes, and we consider that within this altitude range, plants always maintain a growth strategy of carbon storage homeostasis by adjusting NSC content (Yang et al., 2022).

The main influencing factors for Trmmo changes in seedlings from different native altitudes are also different. The Trmmo changes of low-altitude seedlings show a significant positive correlation with leaf morphological structure and Sugar. Studies have shown that this is because leaf morphological structure adjustment changes leaf stomata and water conduction, and plants use sugar osmotic adjustment to maintain turgor, thereby regulating Trmmo changes when low-altitude seedlings are transplanted across different altitude environments (Brun, 1965; Koroleva et al., 2002; Pereira et al., 2022). This is consistent with our findings. Notably, the regulatory effects of leaf morphological structures such as AvgPA and SLA on Trmmo become nonsignificant when high-altitude seedlings are transplanted across different altitude environments. We propose that this phenomenon arises from the distinct mechanism by which these structures mediate Trmmo changes in high-altitude seedlings, and the adaptive advantages conferred by these structures to low-altitude seedlings during transplantation across different altitude environments are not applicable to high-altitude seedlings under such transplantation scenarios (Manishimwe et al., 2022; Zhou J. et al., 2023). This experiment further confirms that high-altitude seedlings turn to carbon storage substances as the dominant factor, and NSC is the only significant factor. This transformation confirms that plants in high-altitude environments give priority to ensuring survival rather than adjusting leaf morphological structure to affect Trmmo changes (Zhou Q. et al., 2023).

However, new branches did not show a significant influence on photosynthetic and Trmmo changes in both types of seedlings, which indicates that the resource allocation tendency in the seedling stage tends to favor building a leaf structure system rather than branch morphological formation. This is in line with the growth law that plants give priority to ensuring basic physiological functions in the seedling stage (Sun et al., 2019; Tang et al., 2022; Xiang et al., 2009). Through random forest analysis, the differences in influencing factors of physiological functions between the two provenances reveal the growth path differentiation caused by native altitude training (Kieltyk, 2024; Kitagawa et al., 2018). Low-altitude seedlings significantly affect photo and Trmmo through leaf morphology and carbon storage substances, which is in line with the path of morphological regulation of physiological functions in Hypothesis 3); high-altitude seedlings are dominated by nutrient factors such as the C/N ratio and NSC in photo and Trmmo changes, verifying the inference of nutrient regulation of physiological functions. Notably, Hypothesis 3) does not involve the common influence of carbon storage substances on the two provenances, and does not consider the weakening influence of leaf morphological structure on Trmmo in high-altitude environments, which is supplemented in our study.

### 4.4 Path differences of *Picea asperata* from different native altitudes

Path analysis results showed that the direct effect of transplantation altitude on the morphological traits of low-altitude seedlings did not reach a significant level, indicating that the change in transplantation altitude is not the dominant factor affecting their morphology (Jin and Qian, 2023). However, morphological traits exerted an extremely significant positive predictive effect on nutrient traits and also had a significant direct effect on physiological functions (Rascher et al., 2004; Sonawane et al., 2021). This indicates that the changes in the morphological characteristics of low-altitude seedlings may be a core component in environmental changes, governing the nutrient absorption and physiological functions of plants (Adams and Kolb, 2004; Guha et al., 2010). Through the comparison of path differences, the direct effect of nutrient traits on physiological functions did not reach a significant level. We believe that compared with changes in nutrient traits, the adaptive changes in plant morphological structure may be a more direct and efficient regulatory pathway for low-altitude seedlings to respond to altitude environments (Ares and Fownes, 1999; Shahba and Bauerle, 2009).

According to the path analysis results for high-altitude seedlings, transplantation altitude exerted an extremely significant positive predictive effect on nutrient traits (Wang et al., 2023; Zhao et al., 2014). According to previous studies, it can be determined that altitude environment changes can directly affect nutrient accumulation in high-altitude seedlings, becoming an important influencing factor for material storage, which is consistent with our results (Lin et al., 2022).However, although the direct effect of nutrient traits on morphological traits did not reach a significant level, nutrient traits exerted an extremely significant positive effect on physiological functions; that is, in the environmental changes experienced by high-altitude seedlings, nutrient traits are the core factor affecting their physiological functions (Jia et al., 2021; Jiménez et al., 2013). By comparing path differences, the direct effect of morphological traits on physiological functions was not significant, in contrast to the significant effect of nutrient traits. For this result, we consider that compared with morphological structure adjustment, nutrient indicator changes caused by altitude changes may be a more effective path for physiological function optimization (Scartazza et al., 2014; Sun et al., 2021; Zhang Q. F. et al., 2019).

This shows distinct path differences in adapting to the environment under altitude changes between seedlings from different native altitudes (Hadi et al., 2022; Tarvainen et al., 2022; Walter et al., 2024). Low-altitude and high-altitude seedlings adapt to the environment through the indirect path of morphology-nutrient-physiology and the direct path of nutrient-physiology, respectively, which aligns with the path differentiation presupposed in Hypothesis 3); but Hypothesis 3 does not explain the path-level differences and the direct driving mechanism of transplantation altitude on nutrients in high-altitude provenances, which we have supplemented through PLS-PM path result analysis.

## 5 Conclusion

A comparison of the responses of *Picea asperata* seedlings from two different provenances under different altitude habitats showed that:

1. Native altitude regulated the physiological traits, SLA, and Nmass content of seedlings, but did not affect leaf structure or carbon storage. During the regulation by transplantation altitude, not only were nutrient traits and leaf morphology altered, but carbon storage was also changed.
2. Low-altitude seedlings showed synchronous optimization of physiology, leaf traits, and carbon storage at the transplantation altitude of 1900 m, demonstrating environmental dynamic plasticity; high-altitude seedlings showed advantages in their native environment, but their adaptability decreased across all traits when transplanted to low altitude, demonstrating native environment conditions adaptation.
3. There were differences in the influencing factors of photosynthetic physiological functions between the two provenances. The morphological traits of low-altitude seedlings had significant effects on both nutrient traits and physiological functions, and they indirectly regulated nutrients through morphological changes to affect physiological functions; high-altitude seedlings directly affected physiological functions through nutrient indicator changes.

This study was conducted within the altitude range of 1600–2400 m. The adaptability of *Picea asperata* seedlings at other altitude gradients and the changes in their growth strategies after longer-term environmental conditioning need further research.

## Data Availability

The raw data supporting the conclusions of this article will be made available by the authors, without undue reservation.
